# Characterization of real-world networks through quantum potentials

**DOI:** 10.1371/journal.pone.0254384

**Published:** 2021-07-13

**Authors:** Nicola Amoroso, Loredana Bellantuono, Saverio Pascazio, Alfonso Monaco, Roberto Bellotti

**Affiliations:** 1 Dipartimento di Farmacia—Scienze del Farmaco, Università di Bari, Bari, Italy; 2 Istituto Nazionale di Fisica Nucleare, Sezione di Bari, Bari, Italy; 3 Dipartimento di Scienze Mediche di Base, Neuroscienze e Organi di Senso, Università di Bari, Bari, Italy; 4 Dipartimento Interateneo di Fisica, Università di Bari, Bari, Italy; Unviersity of Burgundy, FRANCE

## Abstract

Network connectivity has been thoroughly investigated in several domains, including physics, neuroscience, and social sciences. This work tackles the possibility of characterizing the topological properties of real-world networks from a quantum-inspired perspective. Starting from the normalized Laplacian of a network, we use a well-defined procedure, based on the dressing transformations, to derive a 1-dimensional Schrödinger-like equation characterized by the same eigenvalues. We investigate the shape and properties of the potential appearing in this equation in simulated small-world and scale-free network ensembles, using measures of fractality. Besides, we employ the proposed framework to compare real-world networks with the Erdős-Rényi, Watts-Strogatz and Barabási-Albert benchmark models. Reconstructed potentials allow to assess to which extent real-world networks approach these models, providing further insight on their formation mechanisms and connectivity properties.

## Introduction

Complex network models are an effective and versatile tool to describe the characteristics of complex systems, consisting of a large number of elementary units interacting with each other, and to study the phenomena underlying their dynamics of operation and evolution [1–4]. Nowadays, the science of complex networks allows the investigation of a large number of aspects of the real world on a multiplicity of sectors and observation scales, ranging from e.g. the interaction mechanisms of our genes, whose malfunction is at the root of the onset of several diseases [5–7], to the competitive dynamics among companies, institutions or even countries in crucial socio-economic contexts such as the search for funding [8–10], the export ecosystem [11, 12] or international rankings [13]. The use of a complex network makes it possible to examine, through a rigorous approach, the structure, efficiency and resilience of the connections between the constituents in the system that it models, whether physical or conceptual. The main tool for analyzing these features is network connectivity, which refers to properties and patterns arising from the topological organization of nodes. Depending on the examined network, peculiar behaviors can occur. For example, in the brain network domain, functional and structural connectivity patterns revealed through neuroimaging techniques yield biomarkers of aging processes and associated pathological conditions [14–21]. Network connectivity is also exploited in several domains, such as Ecology [22, 23], Geomorphology [24], Social Network Science [25] and Systems Biology [26], to characterize a number of collective phenomena [27] and understand the underlying dynamics. In particular, seminal studies have demonstrated how connectivity can be suitably described in terms of the spectral properties of a network such as the spectrum of its Laplacian [28–30].

In a recent work [31], we proposed a quantum-inspired definition of potential energy for complex networks. Using a proper dressing transformation, we reconstructed the potential energy of a network by identifying a Shrödinger-like equation whose spectrum coincides with that of the graph normalized Laplacian. In particular, we demonstrated that such framework detects the onset of collective phenomena such as the emergence of a giant component in the Erdős-Rényi model (ER); besides, we showed that the intrinsic variability of a random network ensemble at fixed connection probability can be measured in terms of the Higuchi fractality of its representative ensemble potential [32].

In the present study, we investigate to which extent potential energy can be adopted to characterize other established network models, specifically the small-world and scale-free ones. Small-world topologies deserve particular attention, since they represent a broad class of networks, among which a relevant role is played by Watts-Strogatz models [33]. Besides, scale-free networks [34] are interesting as well, since they have been suitably used in many real-world applications [35–37]. Despite their simplistic nature, these models are able to capture peculiar patterns and behaviors of real-world networks. Therefore, here we intend to provide a comprehensive analysis of potential energy associated to these popular network models and show how the proposed quantum-inspired approach yields further insight on their well-known properties. Thus, one goal of this work is to extend our comprehension about the behavior of potential energy when associated to random, small-world and scale-free networks. Then, based on this knowledge, we focus on several real-world networks, characterized by different size and topology and generally featuring heterogeneous connectivity patterns, in order to show how potentials can shed light on the differences among them. As we shall discuss in the following, the proposed framework represents a useful tool to investigate connectivity and unveil the underlying dynamics of real-world networks, providing a new method to compare them with benchmark network models.

The article is organized as follows: in the “Materials and methods” section, we provide a short overview about the Laplacian of a network and the methodology adopted to reconstruct the potential associated with its spectrum; besides, we briefly present the main characteristics of small-world and scale free networks, especially considering the Watts-Strogatz (WS) [33] and the Barabási-Albert (BA) models [34]. In the “Results and discussion” section, we examine the application of potentials to small-world and scale free networks, then we perform a comparison of real-world networks with ensembles of artificial networks, based on potentials. In the “Conclusion” section we highlight the perspectives of further developments and investigations.

## Materials and methods

**Fig 1:**
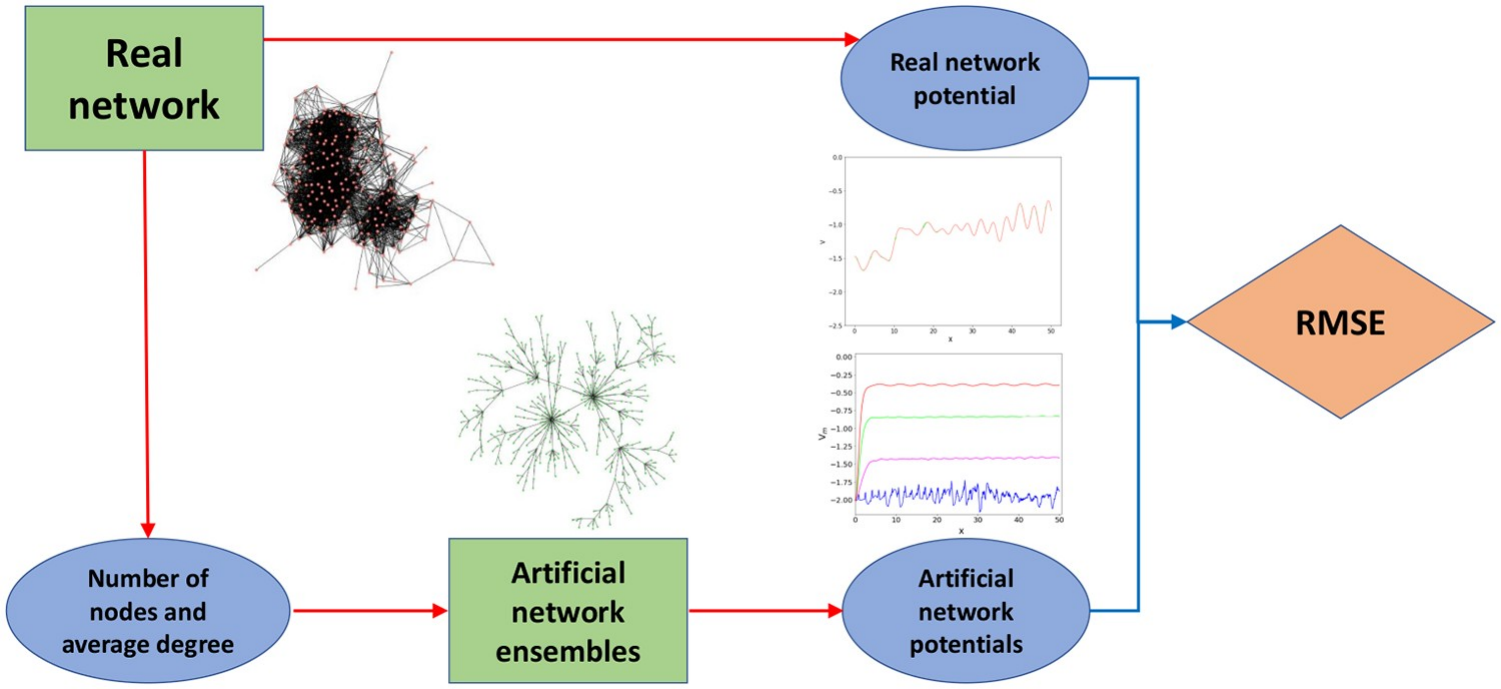
Evaluation of the similarity between a real-world network and different ensembles of artificial networks. The similarity is measured as the average root-mean-square error (RMSE) of the real network potential with respect to the potentials associated with the networks of each ensemble. Moreover, we compare these results with the RMSE between the real network potential and the ensemble median potentials.

In this work, we present a methodology designed to investigate the connectivity of a network by means of a quantum-inspired potential energy (to be called “potential” in the following). Our framework allows the comparison of real-world networks among each other, and their characterization with respect to a set of benchmark models. A comprehensive overview of this pipeline is reported in Fig 1, while details on each step are discussed in the following.

### From the graph spectrum to the graph potential

The definition of a graph G=(N,E) involves a set N of *N* vertices (nodes) and a set E of edges (links) connecting pairs of nodes [38]. A graph is generally represented with an adjacency matrix *A*, whose elements *a*_*ij*_ are not null if *i* and *j* are adjacent to each other, namely if a link (*i*, *j*) between them exists; the strength of this connection can also be quantified by providing the link (*i*, *j*) with a weight *w*_*ij*_. In this work, we consider binary (*a*_*ij*_ = 0, 1) and symmetric (*a*_*ij*_ = *a*_*ji*_) adjacency matrices which represent *unweighted* and *undirected* graphs. For the sake of simplicity, we also consider *a*_*ii*_ = 0 for all nodes, thus excluding loops from the network.

The degree *d*_*i*_ measures the number of connections of the node *i*; in an undirected and unweighted network, it is calculated as the sum of the *i*-th row (or the *i*-th column) of *A*. Considering the diagonal degree matrix *D*, defined by *D*_*ii*_ = *d*_*i*_, it is possible to introduce graph Laplacian *L* = *D* − *A* and its normalized version L:

$$\begin{array}{c} L_{ij}=\{\begin{array}{cc} 1 & \;\text{if}\;i=j\;\text{and}\;d_{i}\neq 0\;\\ -\frac{1}{\sqrt{d_{i}d_{j}}} & \;\text{if}\;i,j\;\text{adjacent}\\ 0 & \;\text{otherwise} \end{array} \end{array}$$ (1)

For disconnected networks, i.e. networks where there exist two or more groups of nodes that cannot be reached from each other by any path, both the Laplacian matrices *L* and L can assume a block-diagonal form in which each block corresponds to a specific component. The Laplacian reveals many interesting properties of a network such as the number and size of its connected components and presence of peculiar structures [28, 39]. From now onward, we will refer to the spectrum of the normalized Laplacian as the *spectrum of the graph*.

Let us sort the eigenvalues λ_*i*_ of the graph spectrum, which are defined in the interval [0, 2], in increasing order so that:

$$\begin{array}{c} 0=\lambda_{1}\leq \lambda_{2}\leq \cdots \leq \lambda_{N}\leq 2, \end{array}$$ (2)

with each eigenvalue counted as many times as its degeneracy. These eigenvalues can be related to the discrete energy levels of a 1−dimensional Schrödinger equation with a specific potential. First of all, we focus on the shifted spectrum:

$$\begin{array}{c} E_{n}=\lambda_{n}-\lambda_{N}\in \left[-2,0\right], \end{array}$$ (3)

then, we construct a network potential *V*(*x*) such that the negative energy levels of the Schrödinger equation

$$\begin{array}{c} -\partial_{x}^{2}\psi \left(x\right)+V\left(x\right)\psi \left(x\right)=E_{n}\psi \left(x\right),\;\text{with}\;\int dx\;{\left|\psi \left(x\right)\right|}^{2}=1, \end{array}$$ (4)

coincide with the negative shifted eigenvalues *E*_*n*_ < 0. The potential *V*(*x*) is built in an iterative process, described in detail in [31] and based on the dressing transformations [40–44]. The workflow starts from the initial guess *V* = 0 and proceeds towards including at each step one more normalized Laplacian eigenvalue as an energy level of the Schrödinger equation. The differential equations used to update the potential at each step are solved by a fourth-order Runge-Kutta (RK) method with step size *h* = 10^−5^, to guarantee high reconstruction accuracy. It is worth noting that the potentials retrieved with this procedure are even by construction.

The reconstruction process provides also the possibility to determine the eigenfunctions of the Schrödinger equation. It would be interesting to find general quantitative relations between their properties and the features of the diffusion modes on the network, to which the Laplacian eigenvectors are physically related. Such an investigation will be the objective of future research.

### Small-world and scale-free networks

In many cases, real-world networks show an interesting behavior: they seem to be characterized by high clustering and low diameter, two properties which highlight the local robustness of the network and the efficiency for long-distance interactions, respectively. The coexistence of both these features in a network is generally known as the small-world effect. If a network with cardinality *N* displays such behavior, the average distance between two randomly chosen nodes scales as ln *N* [2, 38]. The Watts and Strogatz model (WS) [33] has been introduced to provide an accurate description of real networks, that interpolates between those given by regular lattices, featuring a high clustering coefficient, and random networks, characterized by a low diameter.

The WS model depends on three parameters, namely the network size *N*, the average degree *c* and the rewiring probability *p*_*rew*_. The starting point to construct a WS network is a regular lattice of *N* nodes (e.g. a ring), where each node is connected to its neighbors up to a range *r*; therefore the average degree of this initial configuration is *c* = 2*r*. Then, each link is rewired to a randomly chosen node with probability *p*_*rew*_. For small *p*_*rew*_, the network keeps the initial high clustering but the rewiring procedure creates long-range links that drastically decrease the average distance between nodes, yielding the small-world behavior. For *p*_*rew*_ → 1, all links are rewired with high probability and the network turns into a random one.

Among small-world networks, scale-free networks deserve a particular mention. The interest for these networks is twofold: on the one hand, scale-free networks provide a better understanding of the dynamics underlying a particular class of small-world networks; on the other hand, the behavior of important real-world networks is demonstrated to be scale-free more than a general small-world. The most popular model for simulating scale-free networks is the Barabási-Albert (BA) model [34].

The BA model was initially introduced to bridge the gap between random and real networks. Actually, both the Erdős-Rényi and Watts-Strogatz models yield networks in which the degree distribution is peaked around the average degree, with fluctuations of the order $\sqrt{N}$, where *N* represents the number of network nodes. On the other hand, real world networks are typically characterized by power-law deegree distributions, hence featuring very large fluctuations in the degree of their nodes. Barabási and Albert managed to retrieve this feature in their model by a progressive construction of the network, based on *preferential attachment*: a new node is added to the network, forming a fixed number *m* of new connections; the probability for pre-existing nodes to connect to the new one is proportional to their degree. This formation mechanism is able to explain the dynamics behind many real-world network organizations.

### Measure of fractality

To quantify the variability of the potentials reconstructed from different networks sampled out of a given ensemble, we introduce an *ensemble potential*, that will be defined in detail in the following section, and characterize its fractal properties. The results in Ref. [31] show that the ensemble potential associated to ER network models with different connection probabilities *p* has a pronounced fractal behavior close to the critical value *p*_*c*_ = 1/(*N* − 1). In the network ensembles we consider in this paper, there is no expected phase transition; however, we are interested in determining a possible increase of fractality when the average degree becomes closer to 1, corresponding to the value at the ER phase transition. As a fractality measure, we use the Higuchi Fractal Dimension [32]. Given a sequence of values {*V*_1_, *V*_2_, …, *V*_*f*_} (in our case, the values of the ensemble potential), it is possible to extract the subset:

$$\begin{array}{c} \{V_{i},V_{i+k},\ldots ,V_{i+nk}\}, \end{array}$$ (5)

where *i* = 1, 2, …, *k* and *n* is the number of intervals of fixed width *k* contained in the range [*i*, *f*]. Then, we consider the quantities:

$$\begin{array}{c} L_{i}\left(k\right)=\frac{f-1}{n}\sum_{j=1}^{n}\left|V_{i+jk}-V_{i+(j-1)k}\right|, \end{array}$$ (6)

representing the normalized measures of the mean distance between neighboring values in the subset (5). Thus, averaging over all the possible initial points *i*, we obtain

$$\begin{array}{c} \left\langle L\left(k\right)\right\rangle =\frac{1}{k}\sum_{i=1}^{k}L_{i}\left(k\right) \end{array}$$ (7)

Finally, if 〈*L*(*k*)〉 ∼ *k*^−*D*^, *D* is the Higuchi Fractal Dimension (HFD) of the series {*V*_1_, *V*_2_, …, *V*_*f*_}. Since this dependence generally holds within a given range of *k*, we investigate it and compute *D* in the interval 2 ≤ *k* ≤ 800.

## Results and discussion

### Watts-Strogatz networks

**Fig 2:**
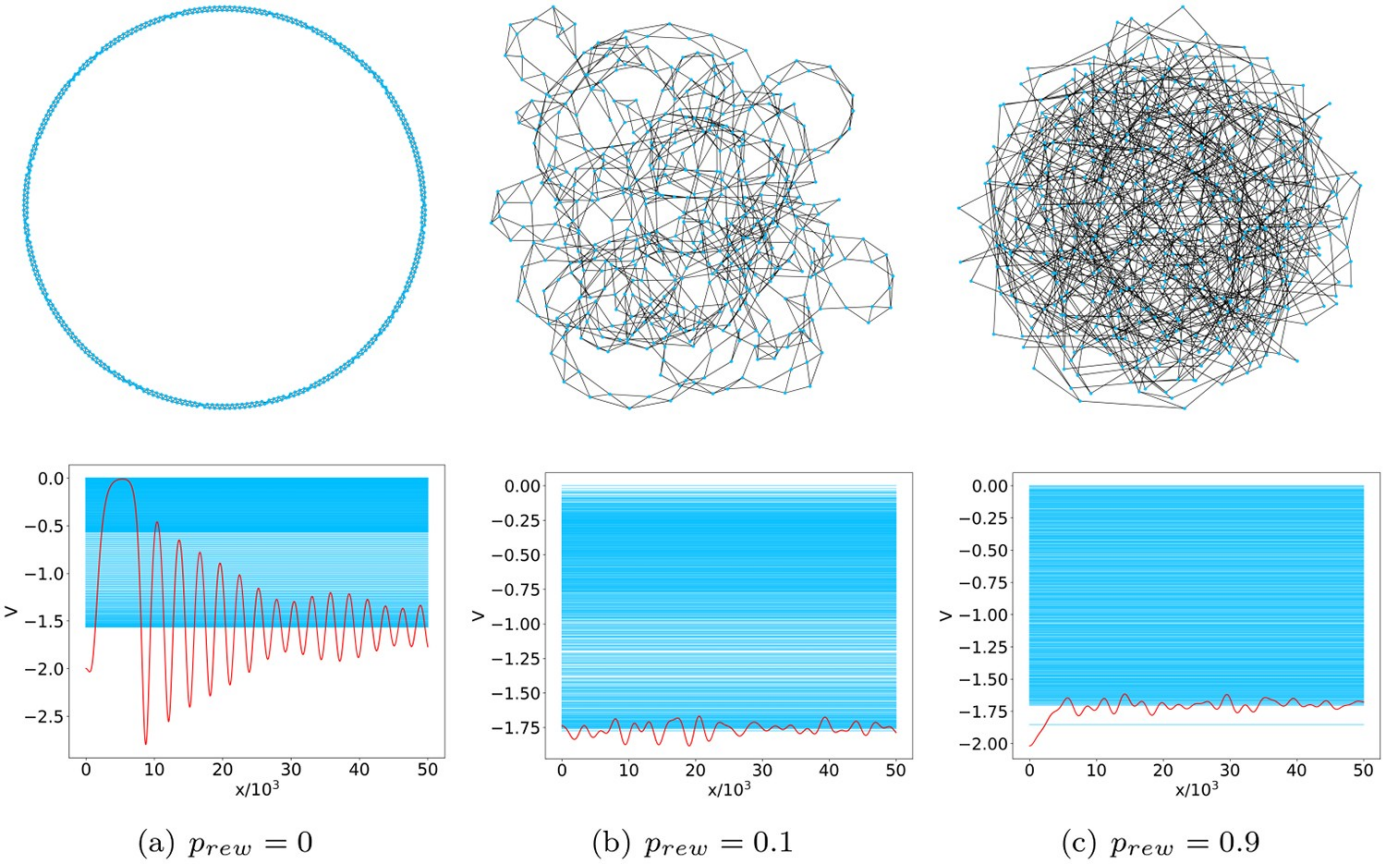
WS models with *N* = 500 nodes and the related reconstructed potentials. The figure shows how topological variability (upper panel), induced in a WS model with *c* = 4 by changing the rewiring probability, is captured by the reconstructed potentials (lower panel). Potentials (red) are superimposed to the network spectrum (pale blue). Notice that the potentials are even by construction.

In this section we show the results obtained by investigating the small world WS model using the network potential approach. In particular, we consider ensembles of WS network realizations with a fixed number of nodes (*N* = 500) and examine how the reconstructed potentials are affected by changes in the average degree *c* and rewiring probability *p*_*rew*_. As the reconstructed potentials are even by construction, their profile will be analyzed in the following by focusing on the positive arguments. Fig 2 displays the structure of WS networks with average degree *c* = 4, for different values of the rewiring probability, together with the associated graph spectrum and reconstructed potential; the analogous outcomes for WS networks with lower (*c* = 2) and higher (*c* = 50) average degree are reported in S1 Appendix.

**Fig 3:**
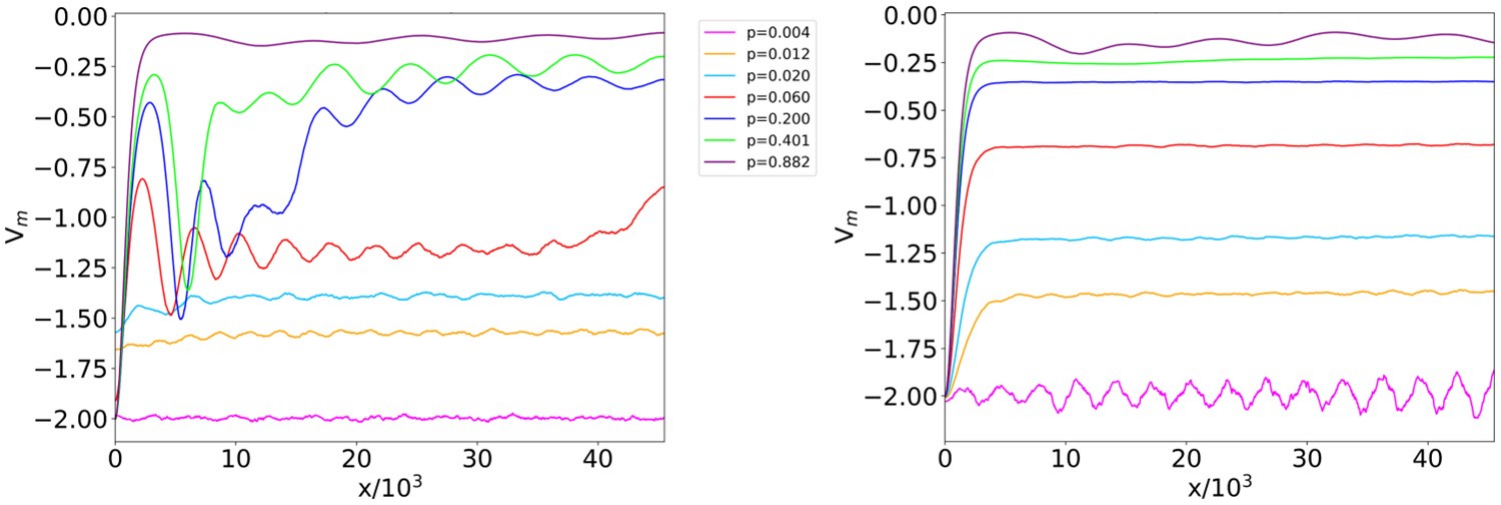
WS ensemble potential at different (*c*, *p*_*rew*_) parameter configurations. Ensemble potentials *V*_*m*_ computed over 100 WS networks with *N* = 500 nodes and rewiring probabilities *p*_*rew*_ = 0.1 (left) and 0.9 (right). To facilitate comparison with the analogous ensemble potentials in the ER network model [31], plots are labeled using the equivalent connection probability *p*, related with the average degree *c* as *p* = *c*/(*N* −1).

As the WS network realizations are constructed through a pipeline which is not entirely deterministic, it is worth focusing on an ensemble potential which is representative of the whole cohort of graphs generated using the WS model at fixed average degree *c* and connection probability *p*_*rew*_. Following the approach developed in [31], we define the WS *ensemble potential* *V*_*m*_ for a given parameter configuration (*c*, *p*_*rew*_) as the pointwise median computed over the set of potentials associated with single-network realizations. Fig 3 shows the profile of the WS ensemble potential, representative of 100 networks generated using the same (*c*, *p*_*rew*_) values, for different configurations of such parameters. Furthermore, to facilitate the comparison between the WS ensemble potential and its counterpart in the ER case [31], information on the average degree *c* is reported in Fig 3 in terms of the equivalent connection probability *p* = *c*/(*N* − 1) in an ER model.

**Fig 4:**
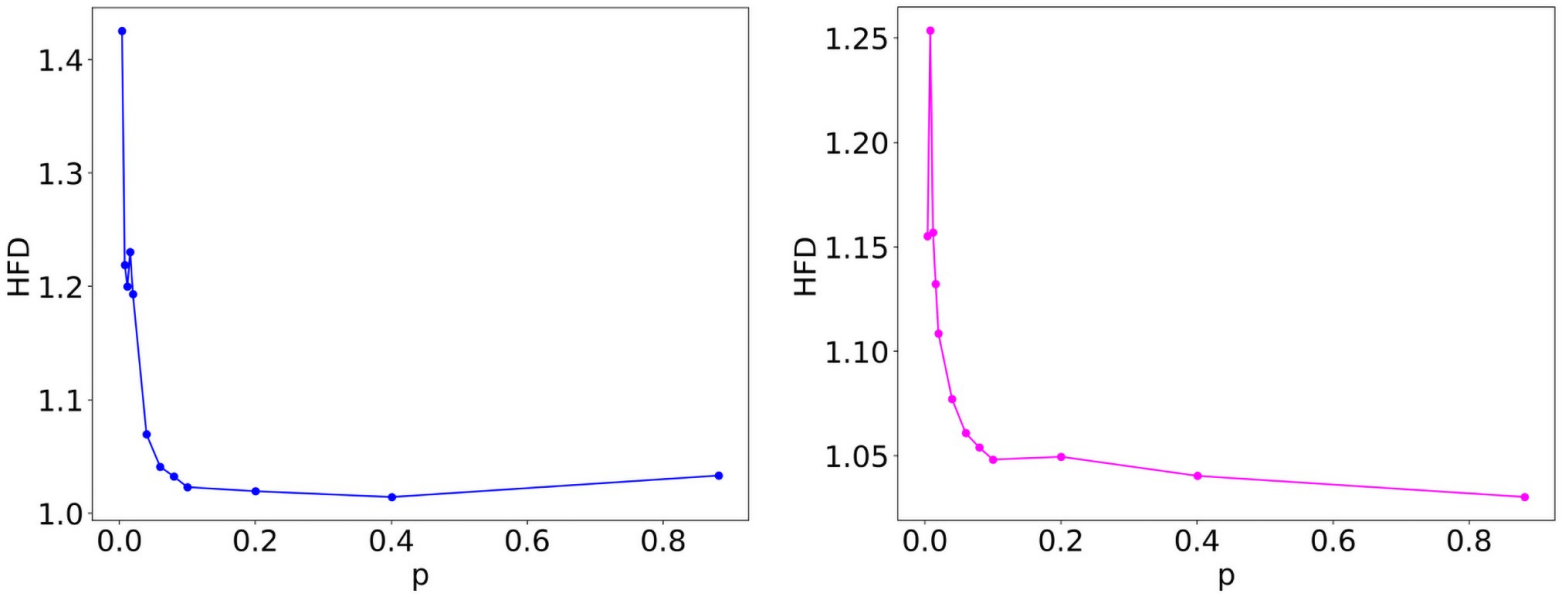
HFD of WS ensemble potentials. Higuchi fractal dimension (HFD) of the ensemble potential *V*_*m*_ over 100 graphs with *N* = 500 nodes and average degree *c*, resulting from a WS model with rewiring probability *p*_*rew*_ = 0.1 (left) and 0.9 (right), as a function of the equivalent connection probability *p* = *c*/(*N* − 1).

Furthermore, to assess the intrinsic variability among the network topologies in the WS ensembles, we compute the HFD of the ensemble potential *V*_*m*_ over 100 WS graphs with the same *c* and *p*_*rew*_. Fig 4 reports the fractal dimension of the WS ensemble potential as a function of the average degree *c* (actually, using the equivalent connection probability *p*), for low (0.1) and high (0.9) values of *p*_*rew*_. Both cases show a substantially stable plateau for connection probabilities larger than 0.1. For connection probabilities approaching 0, fractality increases towards HFD ≃1.43 when *p*_*rew*_ = 0.1 and HFD ≃1.26 for *p*_*rew*_ = 0.9. Unlike the ER ensemble case [31], in which the HFD of ensemble potentials is characterized by a sharp peak around the critical connection probability *p* = 1/(*N* − 1), surrounded by values very close to 1, here we do not observe a peak with these features in the HFD. This result is related to the fact that all the considered WS networks are supercritical by construction, since their average degree *c* (representing the number of neighbors of a node in the initial ring topology) has to be even and thus never reaches the critical value 〈*k*〉 = 1. However, the aforementioned rapid increase of the HFD in Fig 4 occurs as the average degree *c* becomes closer to the value corresponding to the ER phase transition, indicating a remnant of criticality.

**Fig 5:**
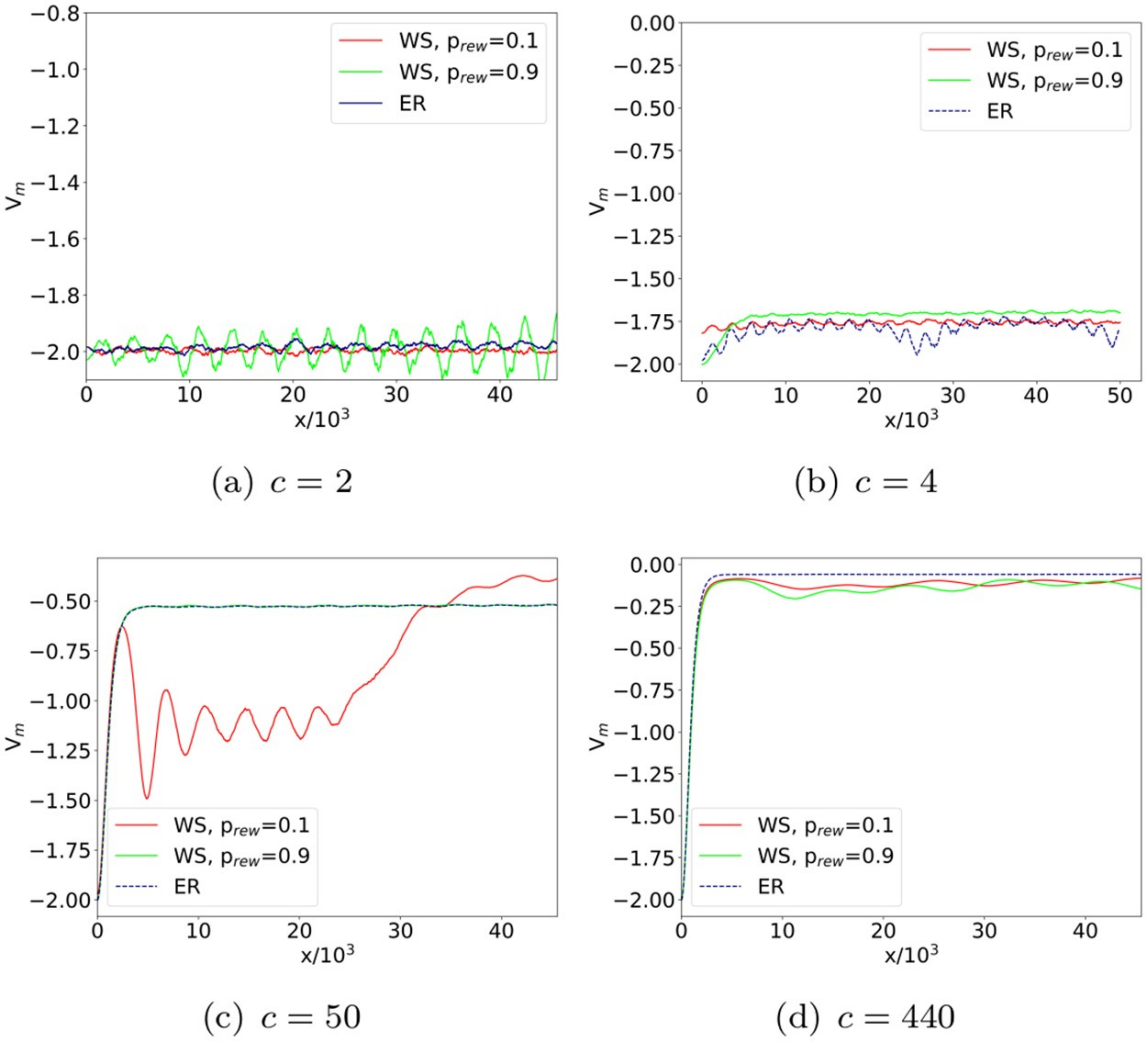
WS and ER ensemble potentials. Direct comparison of ensemble potentials *V*_*m*_ reconstructed from 100 realizations of WS and ER networks with the same average degree *c*. Remarkable discrepancies between the two models emerge at intermediate values of *c*, provided that the rewiring probability of the WS construction is sufficiently small.

It is worth noting that the onset of the small-world behavior follows from a second order phase transition at *p*_*rew*_ = 0 [45]. On the other hand, when *p*_*rew*_ is far from zero, it is possible to recover in the WS model the behavior of random networks with the same average degree (〈*k*〉 = *c*). For a direct comparison, we investigate the discrepancies between WS and ER models in configurations characterized by the same average degree *c* (or, equivalently, the same connection probability *p* = *c*/(*N* − 1)). In fact, according to the meaning of rewiring, one should expect that the higher *p*_*rew*_, the more the randomness injected in the model. Fig 5 displays the comparison between the ensemble potentials of WS networks with average degree *c* = 2, 10, 50, 440, and the ones of ER networks with corresponding connection probabilities *p* = 0.004, 0.02, 0.1, 0.9.

As expected, WS ensemble potentials of networks with *p*_*rew*_ = 0.9 tend to better reproduce the behavior of their ER counterparts than in the low-*p*_*rew*_ case. Interestingly, we notice a discrepancy between WS and ER ensemble potentials at intermediate values of the average degree *c*. On the other hand, in the case *c* = 2, WS and ER networks are both characterized by a large component with sparse connections, a configuration corresponding to a small clustering coefficient. In the case *c* = 440, instead, both networks correspond to almost complete graphs, whose dense connections yield large clustering coefficients and relevant small-world effect.

### Barabási-Albert networks

**Fig 6:**
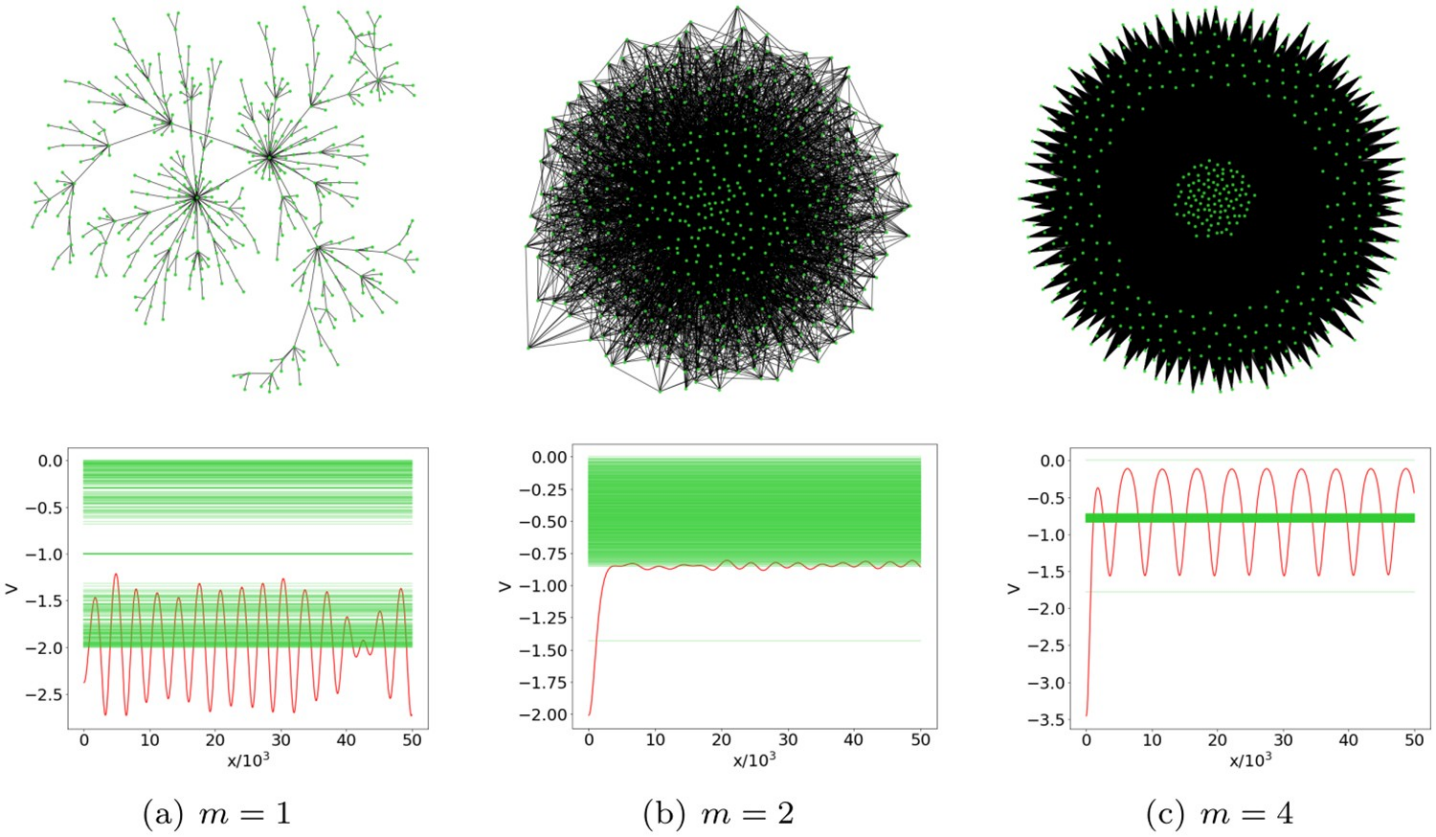
BA models with *N* = 500 nodes and the related reconstructed potentials. The figure shows in the upper panel three examples of BA networks (*m* = 1, 10, 400) while in the lower panel the reconstructed potentials (red) are superimposed to the corresponding graph spectrum (green).

Among the small-world networks, we consider for further insight the potentials associated with the particular case of scale-free networks, obtained through the BA formation mechanism. The only parameter of this model is the number *m* of pre-existing nodes to which a newly-added node is connected. The construction of a BA network generates in the limit *N* → ∞ a degree distribution characterized by a *k*^−3^ behavior for large *k* [2, 34], which determines a finite average degree, equal to 2*m*, but a divergent variance. Notice that, for finite-size BA networks, the estimate 〈*k*〉 = 2*m* is valid only if *m* ≪ *N*. In the following, we shall focus on BA models with *N* = 500 nodes and *m* ∈ [1, 400]. Since for *m* = 1 the average degree 〈*k*〉 = 2 is larger than 1, all the networks are supercritical, as in the WS case; some examples of BA networks and their respective reconstructed potentials are shown in Fig 6.

**Fig 7:**
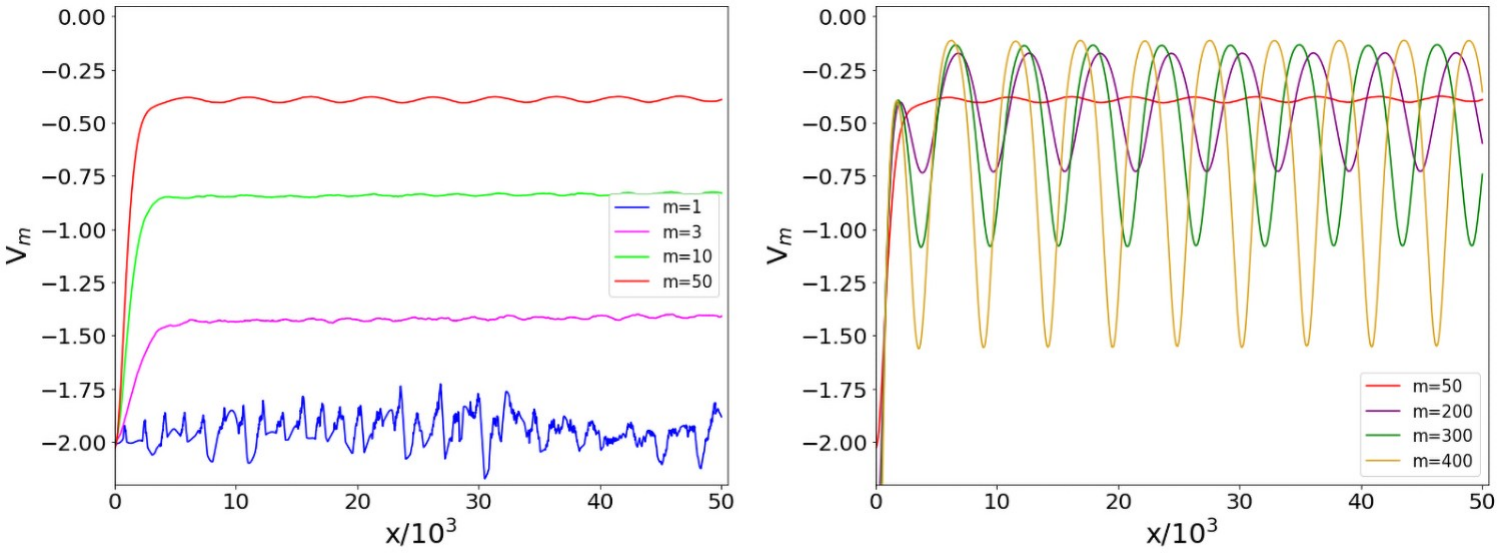
BA ensemble potential for different *m* values. Ensemble potentials *V*_*m*_ computed over 100 BA networks with *N* = 500 nodes and *m* connections made by each new node to the existing ones in the graph construction procedure. The figure shows how potentials vary according to the parameter *m* ranging from 1 to 400, with the left (right) panel reporting the cases of smaller (larger) *m*.

The profiles of the ensemble potentials, computed over 100 BA network realizations at fixed *m*, are displayed in Fig 7. Though we did not expect to observe criticality, we detect more evident ruggedness of the potential as the limit value *m* = 1 is approached. Increasing *m*, the curves become smoother, resembling the supercritical ensemble potentials of the ER case [31]. However, for *m* beyond 50, the periodic oscillations on top of the BA ensemble potential plateau became more and more relevant, marking a significant deviation from the behavior of ER random networks.

**Fig 8:**
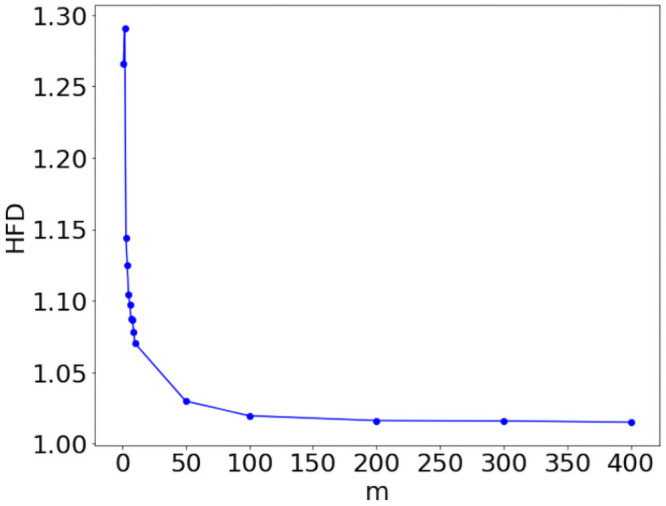
HFD of BA ensemble potentials. Higuchi fractal dimension (HFD) of the ensemble potential *V*_*m*_ over 100 graphs with *N* = 500 nodes, resulting from a BA model, as a function of the parameter *m*.

The qualitative observation that ruggedness increases by approaching criticality from above is confirmed by the Higuchi fractal dimension computed for the analyzed BA network ensembles, whose profile as a function of *m* is displayed in Fig 8. The HFD reaches values between 1.25 and 1.30 for *m* = 1 and 2, and rapidly decreases at larger *m*: the value at *m* = 50 is around 1.02. This behavior closely resembles the one observed in the case of WS networks, confirming that, even for network ensembles that are supercritical by construction, fractality increases as the average degree becomes closer to 1, while it approaches 1 for denser networks.

Following a path started in Ref. [31], we used generative models of artificial networks as benchmarks, to show that the findings derived from the reconstructed potential formalism are consistent with well-established results. Such an operation constitutes a test of the reliability of our approach in detecting network properties, in particular the nontrivial ones, such as the presence of a phase transition (in the case of ER networks discussed in [31]) or the remnants of criticality (in the cases of WS and BA networks discussed above). These results motivate us to employ the reconstructed potentials to unveil the features of real networks.

### Real world networks

The reconstructed potential formalism, used in the previous sections to characterize ensembles of artificial networks, will be now applied to the description of real-world complex systems. In particular, we will compare the potentials associated to real-world unweighted and undirected networks with benchmark potentials derived from test ensembles of artificial networks. The data analyzed in this section are taken from public databases [46, 47] and belong to different domains: animal relationships (free-ranging grey kangaroos [48] and Grévy’s zebras [49]), human interactions (collaborations between Jazz musicians [50] and contacts between suspected terrorists implicated in the Madrid train bombing of 2004 [51]), infrastructures (the European road network [52] and the US power grid [33]) and scientific co-authorship (papers on Astrophysics and High-Energy Physics [53]). The relevant features of these networks are reported in Table 1.

**Table 1:** Relevant features of the real-world networks of different domains used to develop a similarity criterion based on the method of reconstructed potentials. *N*, *L* and *N*_*c*_ denote the number of nodes, edges and connected components, respectively; LCC stands for Largest Connected Component; 〈*k*〉 is the average degree of the network; the fill is defined as the ratio between the number of edges *L* and the total number of possible edges (*N*(*N* − 1)/2 in an undirected network without loops). All data on these networks are available at [46, 47].

Network		Properties					
Name	Domain	*N*	*L*	*N*_*c*_	Size of LCC	〈*k*〉	Fill
Kangaroo	Animal	17	91	1	17	10.71	0.67
Zebra	Animal	27	111	2	23	8.22	0.32
Jazz	Human interactions	198	2742	1	198	27.70	0.14
Train bombing	Human interactions	64	243	1	64	7.59	0.12
EU roads	Infrastructure	1174	1417	26	1039	2.41	2.06 ⋅ 10^−3^
US power grid	Infrastructure	4941	6594	1	4941	2.67	5.40 ⋅ 10^−4^
Astro	Co-authorship	18772	198110	290	17903	21.10	1.12 ⋅ 10^−3^
HeP	Co-authorship	12008	118521	278	11204	19.74	1.64 ⋅ 10^−3^

**Fig 9:**
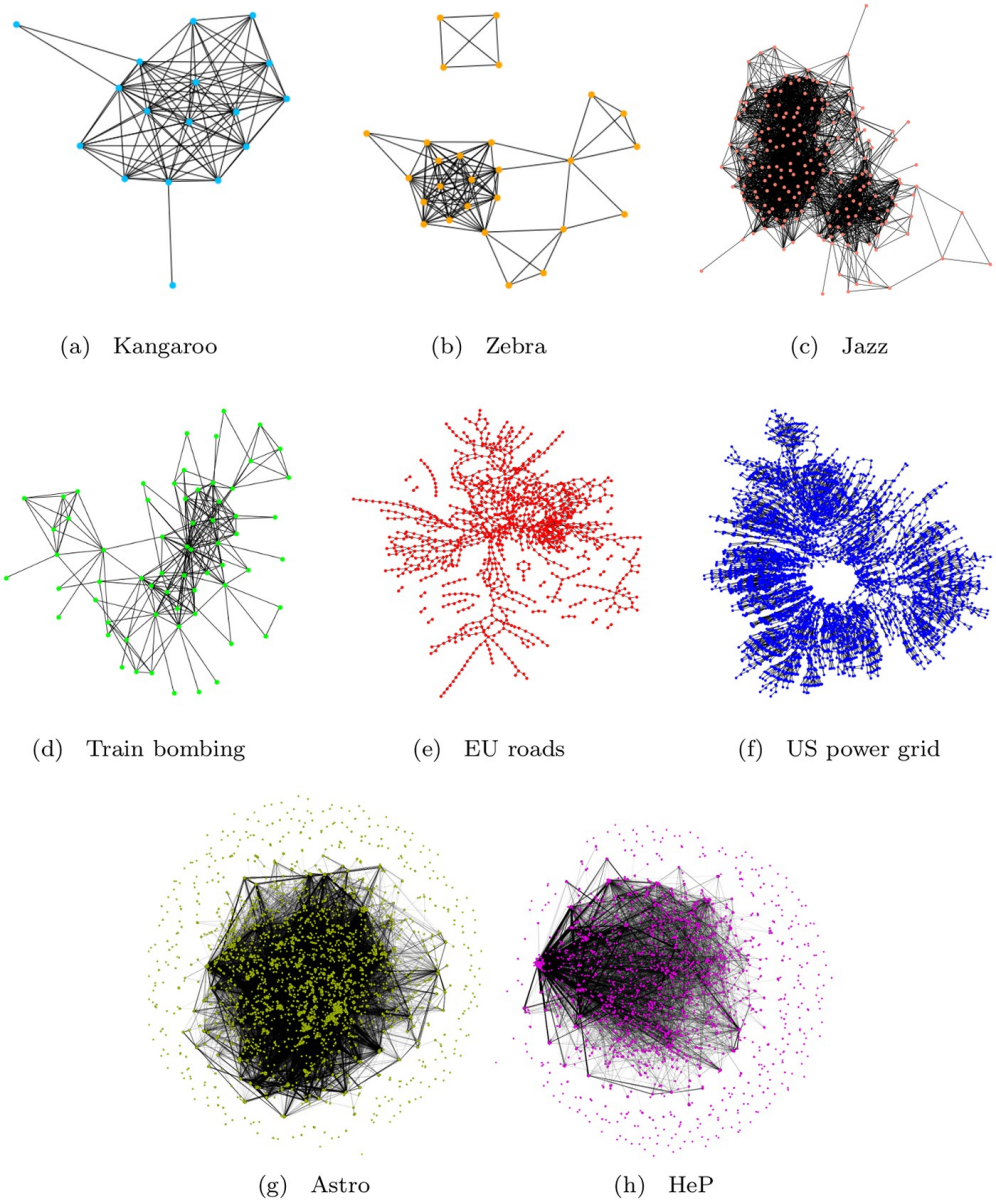
Real-world network graphs. Graph representation of the real-world networks reported in Table 1.

**Fig 10:**
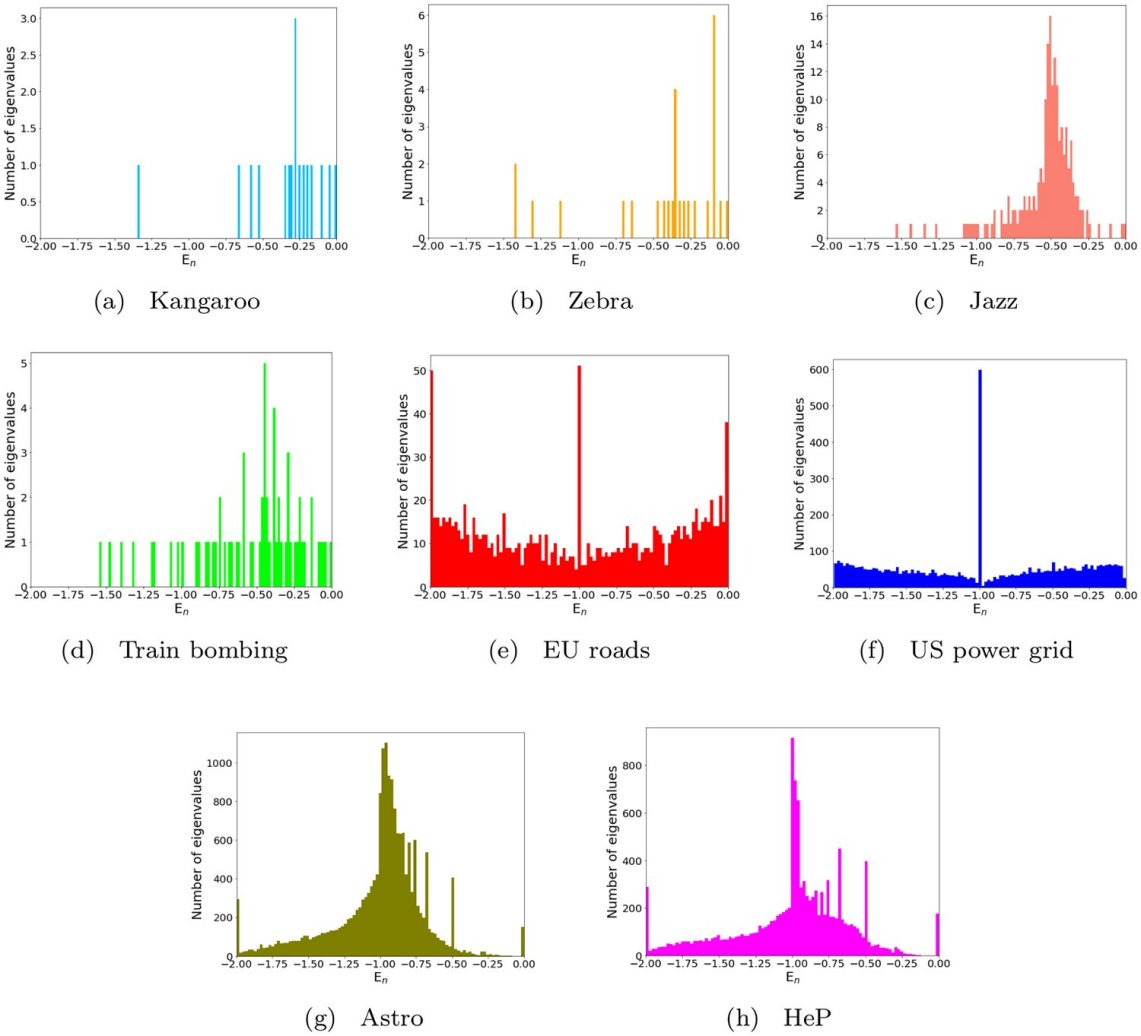
Real-world network spectral distributions. Distributions of graph eigenvalues, shifted to the interval [−2,0], related to the real-world networks reported in Table 1.

**Fig 11:**
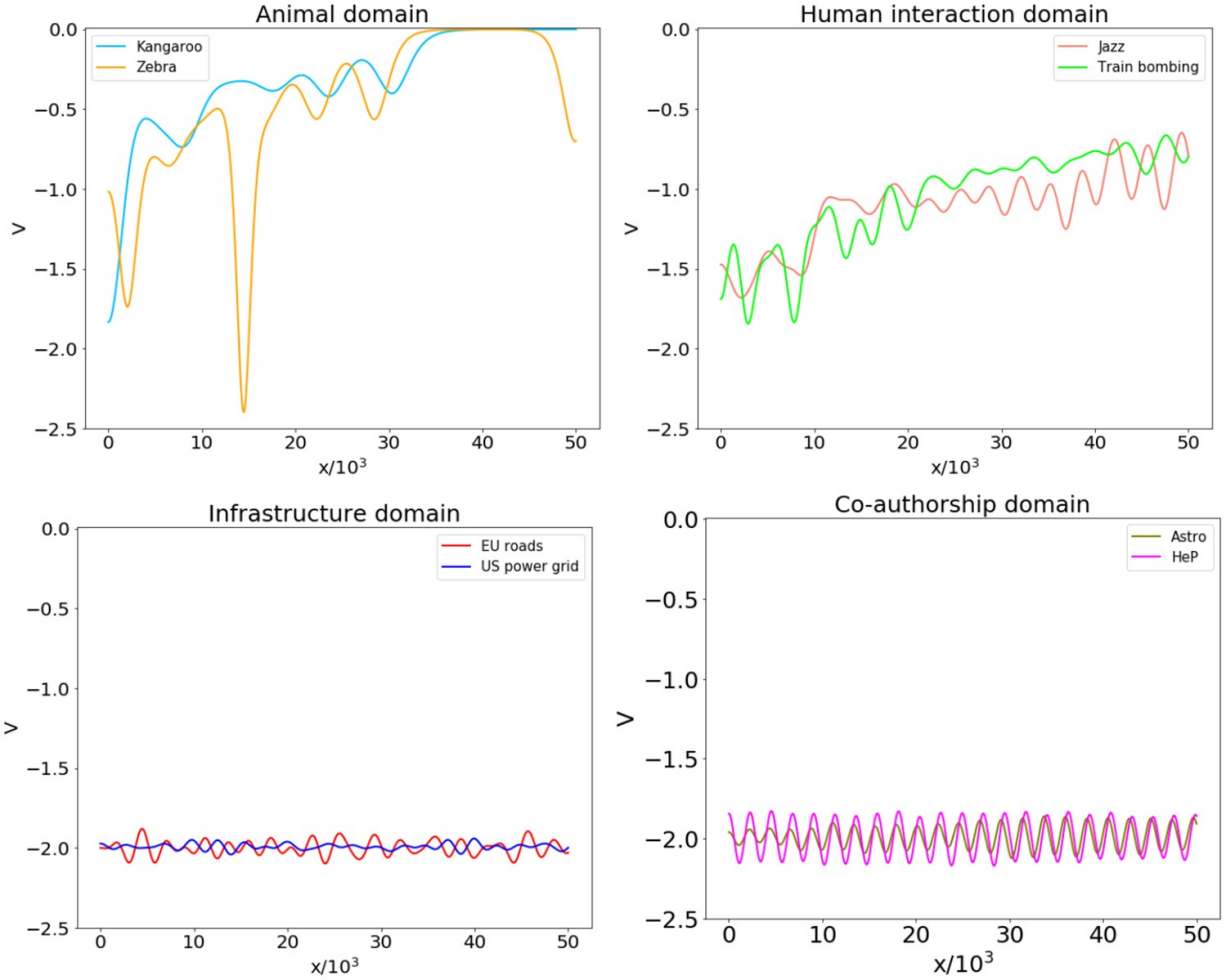
Real-world network potentials. Potentials reconstructed from the graph spectra of the real-world networks reported in Table 1, paired according to their domain.

Fig 9 shows the structure and topologies of the aforementioned real-world networks, while the shifted spectral distribution of their normalized Laplacians are displayed in Fig 10. Finally, the profile of the real-world network potentials, reconstructed from such graph spectra, are reported in Fig 11, where all the plots are presented using the same range to facilitate comparison.

At a first glance, it can be observed that the potentials related to animal and human interactions show an increasing trend in the considered range, while those associated to infrastructures and co-authorship are characterized by oscillations of various amplitudes and do not increase appreciably. Such differences can be related to the structure of the graph spectra. In particular, the potentials with almost no increase are associated to networks whose shifted graph spectra tend to densely fill the interval [−2,0]. Although the considered infrastructure and co-authorship networks share such property, their spectral distributions are qualitatively different: for the former, the eigenvalue density is higher close to the extreme values of the band, while for the latter the eigenvalues close to the extremes are more sparse, being their spectral distribution peaked around −1. This difference reflects in wider and more regular oscillations in the case of co-authorship networks, while the potentials of infrastructures appear more similar to the ones obtained for random networks at slightly supercritical connection probabilities [31]. Actually, the EU roads and US power grid networks are characterized by average degrees 2.41 and 2.67, respectively, while the values for co-authorship networks are larger by one order of magnitude.

For the considered networks describing animal and human interactions, characterized by increasing potentials, the graph spectra consist of an emergent upper band and few smaller isolated eigenvalues. Qualitatively, the low-energy parts of such potentials feature as many wells as the number of such isolated eigenvalues, including degeneracies [31]. For example, in the case of the zebra network, the eigenvalues λ_1_ = λ_2_ = 0 (doubly degenerate, since the network has two components), λ_2_ = 0.126 and λ_3_ = 0.317 correspond to four potential wells, two of which are visible in Fig 11, while the remaining two are on the negative *x* axis in symmetric positions.

After discussing the properties of the real-world networks and reconstructed potentials under investigation, let us focus on the benchmark ensembles to which they will be compared. First, we compute the average degree *k*_*real*_ of the real network we are considering, and derive the reconstructed potential from its normalized Laplacian spectrum. Then, we generate ensembles of artificial networks characterized by the same number of nodes and roughly the same average degree *k*_*real*_ as the real one, according to the following rules:

- An ensemble of 100 ER networks is generated with connection probability *p* = *k*_*real*_/(*N* − 1).
- Three ensembles of 100 WS networks each, respectively characterized by low (*p*_*rew*_ = 0.1), medium (*p*_*rew*_ = 0.5) and high (*p*_*rew*_ = 0.9) rewiring probability, are generated with average degree *c* corresponding to the closest even integer to *k*_*real*_.
- An ensemble of 100 BA networks is generated, with the number of connections *m* made by each newly-added node coinciding with the closest integer to *k*_*real*_/2.

We derive the ensemble potential for each of the aforementioned sets. We do not perform comparison between the animal behavior networks and the BA ensemble, since these are high-fill networks, with no proper sign of preferential attachment, for which there is no analytic estimate of the average degree in terms of *m*.

The quantitative comparison between the real-world networks and the 5 reference ensembles is made by regarding each reconstructed potential associated with a single-network realizations in a given artificial ensemble as a tentative fit of the corresponding real-world network potential. Specifically, the discrepancy between benchmark and real-world potentials is quantified as the related root-mean-square error (RMSE), which constitutes a measure of the affinity between the networks. The results of such comparison, for all the benchmark ensembles under investigation, are shown in Table 2.

**Table 2:** Comparison between the reconstructed potentials of real and synthetic networks. For each real network, we consider five different ensembles of synthetic networks with approximately the same average degree, sampling 100 realizations of each ensemble. We construct a distribution of RMSE between the potentials associated to the real network and to each syntetic network. Mean and standard deviation of each distribution are reported in the table, with network models closest to the real ones highlighted in boldface. The number in brackets represents the RMSE between the real network potential and the ensemble median potential.

Network	RMSE with benchmark networks				
	ER	WS, *p*_*rew*_ = 0.1	WS, *p*_*rew*_ = 0.5	WS, *p*_*rew*_ = 0.9	BA
Kangaroo	0.143±0.022	**0.129** **±** **0.023**	0.146±0.022	0.142±0.025	−
	(0.110)	(**0.098**)	(0.119)	(0.116)	−
Zebra	0.470±0.029	**0.429** **±** **0.018**	0.459±0.029	0.476±0.030	−
	(0.456)	(**0.417**)	(0.444)	(0.464)	−
Jazz	0.472±0.008	0.477±0.007	**0.412** **±** **0.006**	0.476±0.006	0.454±0.007
	(0.472)	(0.475)	(**0.411**)	(0.475)	(0.453)
Train bombing	0.253±0.018	**0.200** **±** **0.024**	0.211±0.016	0.243±0.010	0.243±0.012
	(0.236)	(**0.183**)	(0.193)	(0.230)	(0.229)
EU roads	**0.064** **±** **0.007**	0.068±0.009	0.105±0.023	0.125±0.024	0.374±0.028
	(0.052)	(**0.047**)	(0.048)	(0.048)	(0.232)
US power grid	0.036±0.004	**0.031** **±** **0.004**	0.053±0.014	0.063±0.019	0.370±0.027
	(0.025)	(**0.020**)	(0.021)	(0.022)	(0.222)
Astro	1.122±0.001	**0.735** **±** **0.004**	0.977±0.003	1.138±0.001	1.148±0.001
	(1.122)	(**0.735**)	(0.977)	(1.138)	(1.148)
HeP	1.105±0.001	**0.732** **±** **0.004**	0.959±0.003	1.110±0.001	1.120±0.001
	(1.105)	(**0.732**)	(0.959)	(1.110)	(1.120)

As regards the smallest networks (Kangaroo, Zebra, Jazz, Train Bombing), the RMSE values are very similar to each other, and in many cases network ensembles with the same number of nodes and average degree provide results equal within the error bars. However, the human interaction network potentials are evidently closer to the potentials of WS ensembles, specifically those with *p*_*rew*_ = 0.5 for the Jazz network and both those with *p*_*rew*_ = 0.1 and 0.5 for the Train bombing one. The situation changes in the case of larger networks: for the co-authorship domain, the potentials of WS networks with small rewiring probability are by far the closest to both real networks, while the infrastructure network potentials are apparently best approximated by the potentials of both the ER ensemble and the WS ensemble with *p*_*rew*_ = 0.1. In the latter case, the BA ensemble provides the worst approximation, possibly indicating that preferential attachment is barely relevant in networks in which long-distance connections should be guaranteed, but geographical and economical considerations do not favor the existence of hubs, as opposed, e.g., to the case of air transport.

Another direct method to compare real-world and benchmark networks is provided by the RMSE between the real network potentials and the ensemble potentials associated with each set of artificial networks, computed by means of the pointwise median over potentials of single-network realizations. Results of this comparison are shown in brackets in Table 2. This approach has the advantage of comparing the spectrum of a network with a single representative of the spectral distribution of the ensembles, though it does not provide an estimate of the error in a natural way. The results essentially satisfy a similar hierarchy as that determined through the mediated comparison with the potentials of each network in the ensembles; numerical differences are found only in the case of large ensemble variability, e.g. close to the ER critical point. On the other hand, as shown in the last lines of Table 2, the results obtained for the largest networks are identical to the averages computed with the first method.

## Conclusion

We applied the reconstructed potential framework to analyze two classes of artificial networks, belonging to the Watts-Strogatz and Barabási-Albert ensembles, that are generally more suitable to incorporate the features of real-world networks than the fully random (Erdős-Rényi) ones. By analyzing the ensemble potentials for different values of the model parameters, we found that their fractality, quantified by the Higuchi fractal dimension, takes larger values as the average degree becomes closer to the value corresponding to the ER phase transition, though it cannot be reached by construction. We concluded our analysis by testing the possibility to quantify spectral similarity between networks through the reconstructed potentials. As a case study, we compared the potentials associated to a given real-world network and different ensembles of artificial networks, sharing with the real one the number of nodes and the average degree. Summarizing the main findings, the formalism of reconstructed potentials allowed to identify in a quantitative way a remnant of criticality in artificial networks, and provided the basis to define an instrument of comparison among networks. In particular, the comparison with benchmark ensembles of artificial networks unveiled hidden features and similarities of real-world networks.

The representation of the Laplacian by means of a quantum potential suggests interesting connections with quantum walks on the network [54, 55] and with possible node metrics that can be defined on their basis [56, 57]. We will devote future research to investigating the relation between the reconstructed potential representation and Schrödinger dynamics on a network. Moreover, our results suggest the possibility to define a node centrality, based on the discrepancy between the reconstructed potentials of the network (quantified, e.g, by square-norm) before and after the removal of a specific node. Such a metric would be analogous to the Laplacian centrality [58], that is defined as the relative difference in the sum of the squared eigenvalues due to a node removal. We will investigate in future research possible definitions of potential-based node metrics and their relation with Laplacian centrality and other established node rankings.

A further perspective is represented by the application of the reconstructed potential formalism to characterize the variability of real networks as a consequence of random alterations, such as the random removal of one or more nodes, or the failure of links. By constructing an ensemble consisting of different realizations of the randomizing process of interest, the fractality of the ensemble potential can be used to quantify the sensitivity of the network structure to the considered perturbations.

## Supporting information

S1 Appendix Additional plots of graphs and potentials for WS networks. The S1 Appendix reports plots analogous to Fig 2 for the cases *c* = 2 and *c* = 50.
(PDF)
