# Supplementary material for: Characterization of real-world networks through quantum potentials
Source: PLoS One. 2021 Jul 13;16(7):e0254384. doi: 10.1371/journal.pone.0254384 (PMC8277057; doi:10.1371/journal.pone.0254384)
Supplement: S1 Appendix — The S1 Appendix reports plots analogous to Fig 2 for the cases c = 2 and c = 50. (PDF) [file pone.0254384.s001.pdf]

Characterization of real-world networks through quantum potentials

Nicola Amoroso[1,2◑], Loredana Bellantuono[3◑], Saverio Pascazio[4,2*], Alfonso Monaco[2], Roberto Bellotti[4,2]

1 Dipartimento di Farmacia - Scienze del Farmaco, Università di Bari, Bari, Italy
2 Istituto Nazionale di Fisica Nucleare, Sezione di Bari, Bari, Italy
3 Dipartimento di Scienze Mediche di Base, Neuroscienze e Organi di Senso, Università di Bari, Bari, Italy
4 Dipartimento Interateneo di Fisica, Università di Bari, Bari, Italy

◑These authors contributed equally to this work.
* saverio.pascazio@ba.infn.it

S1 Appendix

Figures 1-2 represent the graphs and reconstructed potentials of Watts-Strogatz (WS) networks with average degree $c = 2$ and $c = 50$, respectively. Both figures show the cases of rewiring probability $p_{rew} = 0, 0.1, 0.9$.

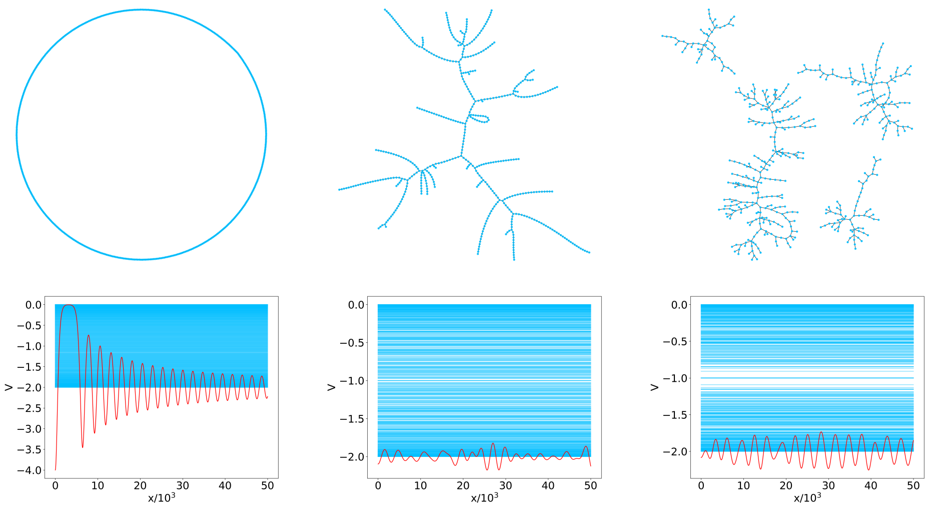

(a) $p_{rew} = 0$ (b) $p_{rew} = 0.1$ (c) $p_{rew} = 0.9$

Figure 1. WS network with $N = 500$ nodes and average degree $c = 2$. Upper panel: graph generated using the WS model with rewiring probability $p_{rew} = 0$ (left), 0.1 (middle) and 0.9 (right). Lower panel: shifted graph spectrum E_n (pale blue horizontal lines) and reconstructed potential $V(x)$ (red) corresponding to the network in the upper panel; since $V(x)$ is an even function, only the positive x semi-axis is displayed.

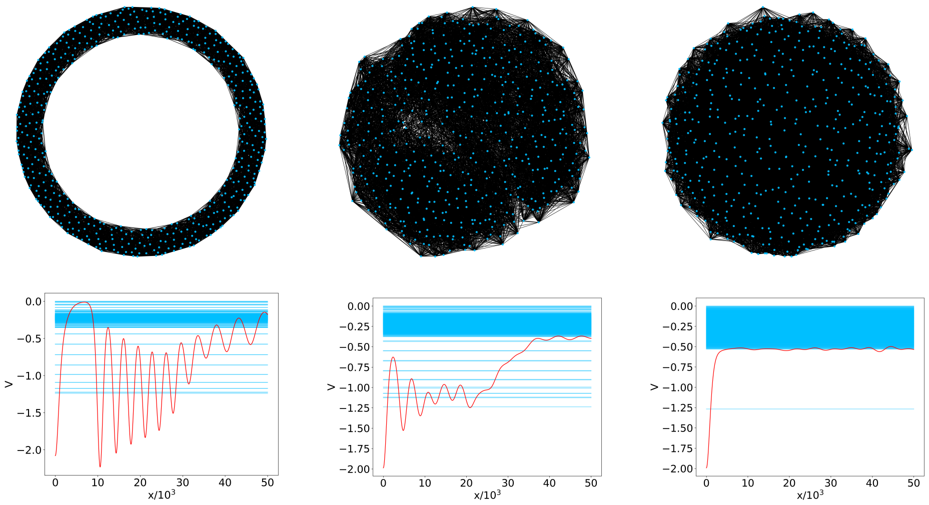

(a) $p_{rew} = 0$ (b) $p_{rew} = 0.1$ (c) $p_{rew} = 0.9$

Figure 2. WS network with $N = 500$ nodes and average degree $c = 50$. Upper panel: graph generated using the WS model with rewiring probability $p_{rew} = 0$ (left), 0.1 (middle) and 0.9 (right). Lower panel: shifted graph spectrum E_n (pale blue horizontal lines) and reconstructed potential $V(x)$ (red) corresponding to the network in the upper panel; since $V(x)$ is an even function, only the positive x semi-axis is displayed.
